# Multilocus disease-causing genomic variations for Mendelian disorders: role of systematic phenotyping and implications on genetic counselling

**DOI:** 10.1038/s41431-021-00933-7

**Published:** 2021-07-19

**Authors:** Dhanya Lakshmi Narayanan, Divya Udyawar, Parneet Kaur, Suvasini Sharma, Narayanaswamy Suresh, Sheela Nampoothiri, Michelle C. do Rosario, Puneeth H. Somashekar, Lakshmi Priya Rao, Neethukrishna Kausthubham, Purvi Majethia, Shruti Pande, Y. Ramesh Bhat, Aroor Shrikiran, Stephanie Bielas, Katta Mohan Girisha, Anju Shukla

**Affiliations:** 1 Department of Medical Genetics, Kasturba Medical College, Manipal, Manipal Academy of Higher Education, Manipal, India; 2 Department of Pediatrics (Neurology division), Lady Hardinge Medical College and Kalawati Saran Children’s Hospital, New Delhi, India; 3 Department of Pediatric Genetics, Amrita Institute of Medical Sciences and Research Centre, Cochin, India; 4 Department of Pediatrics, Kasturba Medical College, Manipal, Manipal Academy of Higher Education, Manipal, India; 5 Department of Human Genetics, University of Michigan Medical School, Ann Arbor, MI USA

**Keywords:** Health care, Genetic testing

## Abstract

Multilocus disease-causing genomic variations (MGVs) and multiple genetic diagnoses (MGDs) are increasingly being recognised in individuals and families with Mendelian disorders. This can be mainly attributed to the widespread use of genomic tests for the evaluation of these disorders. We conducted a retrospective study of families evaluated over the last 6 years at our centre to identify families with MGVs and MGDs. MGVs were observed in fourteen families. We observed five different consequences: (i) individuals with MGVs presenting as blended phenotypes (ii) individuals with MGVs presenting with distinct phenotypes (iii) individuals with MGVs with age-dependent penetrance (iv) individuals with MGVs with one phenotype obscured by another more predominant phenotype (v) two distinct phenotypes in different individuals in families with MGVs. Consanguinity was present in eight (8/14, 57.1%) of them. Thirteen families had two Mendelian disorders and one had three Mendelian disorders. The risk of recurrence of one or more conditions in these families ranged from 25% to 75%. Our findings underline the importance of the role of a clinical geneticist in systematic phenotyping, challenges in genetic counselling and risk estimation in families with MGVs and MGDs, especially in highly inbred populations.

## Introduction

Next-generation sequencing techniques (NGS) like whole-exome sequencing (WES) and whole-genome sequencing (WGS) have become affordable and easily accessible for the diagnosis of Mendelian disorders. The unbiased approach of these techniques has led to significant insights into novel and complex disease-causing molecular mechanisms. The genocentric approaches have also facilitated the elucidation of newer phenotypes other than those established earlier through phenotypic homogeneity. With the integration of these techniques for the diagnosis of rare disorders, the role of a clinical geneticist has evolved beyond clinical evaluation. Molecular and bioinformatics knowledge have become a necessity for contemporary geneticists in order to aid comprehensive elucidation of the phenotypes and their underlying genetic mechanisms [1, 2].

Though the analysis and prioritisation of variants in broad-spectrum tests especially WES is challenging, it provides a unique opportunity to decipher the underlying cause of complex genetic disorders. The use of WES has led to the identification of multilocus disease-causing genomic variations (MGVs) in individuals with multiple genetic diagnoses (MGDs) i.e., more than one genetic diagnosis due to genomic variations at more than one genetic loci, segregating independently [3, 4]. The presence of MGDs in an individual has been reported occasionally as isolated case reports. Recently, there have been reports of a series of individuals with MGDs interrogated retrospectively in either research or diagnostic cohorts. Multiple affected individuals in a single-family, multiple organ system involvement and consanguinity are some factors, which can predict the occurrence of MGVs and MGDs [4, 5]. The incidence of MGDs in individuals diagnosed by WES in different cohorts ranges from 1.4 to 7.2% [3–10].

Identification of MGVs has implications for an individual as well as families with regard to genetic counselling and reproductive choice. We hereby present the complexities of phenotype and genotype of fourteen families with MGVs. We describe how the input of clinical information and systematic phenotyping aided in the dissection of phenotypes, challenges faced in genetic counselling and how MGVs re-defined the risk of recurrence in these families.

## Methods

We conducted a retrospective study of individuals who underwent genetic testing at the medical genetics department in Kasturba Medical College, Manipal, India from January 2015 to March 2021, to identify individuals and/or families with MGVs and analyse the impact of MGVs in such families. Informed consent was obtained from the families for the publication of images and genetic data. The study has the approval of the institutional ethics committee. The probands in families F1, 2, 5, 6, 7, 8, 9, 11, 12 and 13 underwent singleton WES. In F3, in addition to the proband, a sibling also underwent WES. In F4, WES was done for the couple and their offspring. In F14, trio WES was done (proband and parents). WES was carried out as described earlier [11]. Sanger sequencing of *PAX3* and *GJB2* was done for the proband in family 10. The probands in F13 and 14 also underwent chromosomal microarray. Technical details of the genetic tests, method for exome sequencing data processing and variant annotation for the individual families are provided in Supplementary Material. Sanger sequencing was done for confirming the variant and segregating the variants in family members. All the variants and phenotypes were submitted to ClinVar and Leiden Open Variation Database (LOVD). All the families were provided post-test counselling and preconception counselling. The affected individuals in those families were managed by a multidisciplinary approach and supportive care.

## Results

**Fig. 1:**
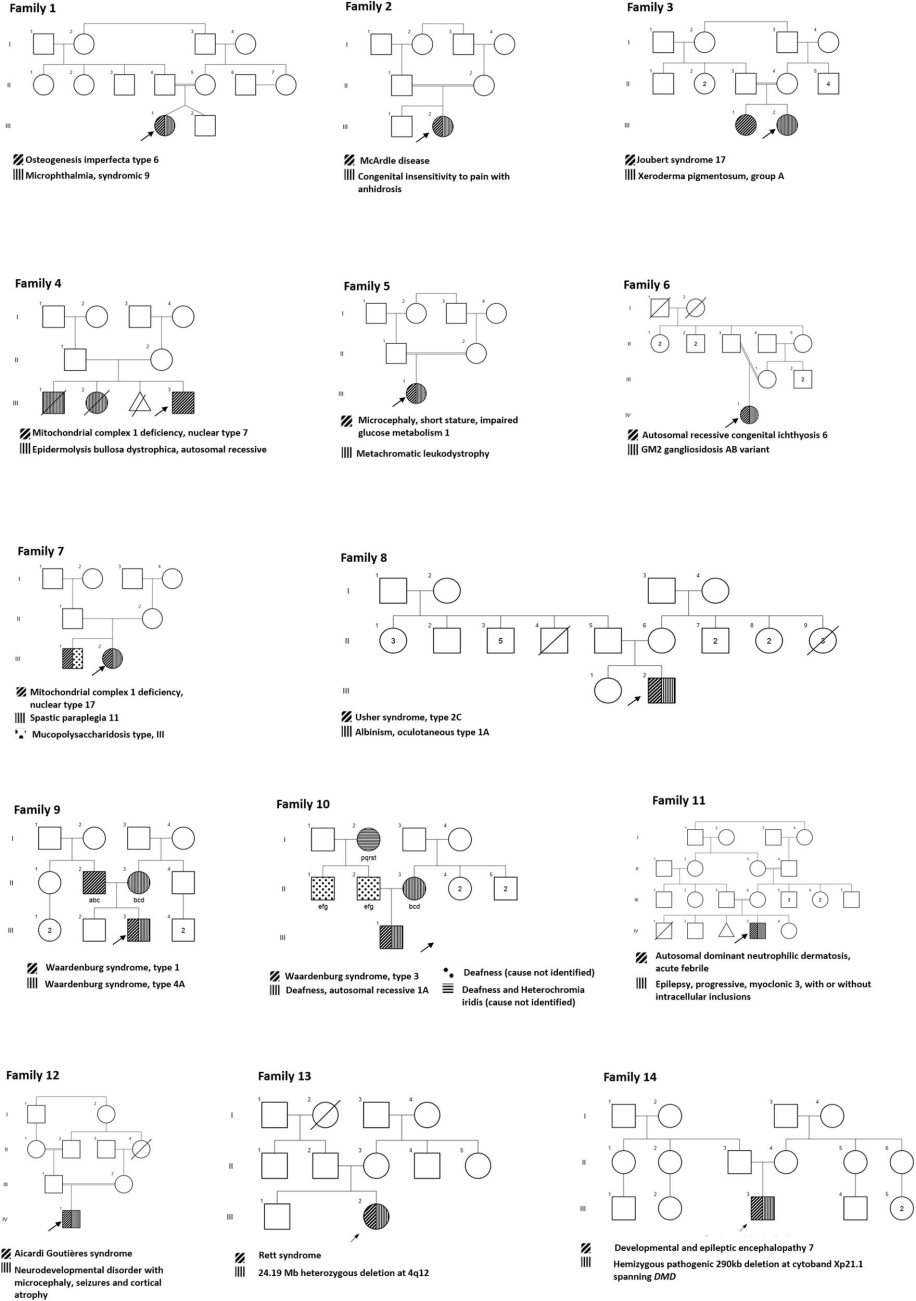
Pedigree of families depicting the affected individuals and their phenotypes.

From January 2015 to March 2021, WES was used for testing 850 families (1158 individuals) and a molecular diagnosis was established in 528 families (528/850, 62.1%). Out of the 528 families who received a molecular diagnosis by WES, we identified thirteen families with MGVs (13/528; 2.4%). Thirteen individuals from 12 families had multiple Mendelian disorders due to MGVs. In two families (F3 and F4), though parents carried MGVs, only a single Mendelian disorder was observed in different individuals in the family. The details of F8, F9 and F10 were published previously [12, 13]. Consanguinity was present in eight (8/14, 57.1%) of them. Thirteen families had two Mendelian disorders and one had three Mendelian disorders. The summary of phenotypic and genotypic characteristics of all the families are enumerated in Table 1. The pedigree of all the families is provided in Fig. 1. Detailed information of affected individuals and families is provided in Supplementary Material.

**Table 1:** Characteristics of families with multilocus disease-causing genomic variants.

Family ID	Consanguinity	Subject ID	Clinical features	Presumed clinical diagnosis	Conditions identified	Gene (Transcript)	Variants^a^	Inheritance pattern	Zygosity
Family 1	Present	III-1	Recurrent fractures, microphthalmia Developmental delay	Osteogenesis imperfecta and Microphthalmia	Osteogenesis imperfecta type VI (MIM# 613982)	*SERPINF1 (NM_ 002615.7)*	c.838_839delCT p.(Leu280Glufs^a^20)	AR	Hom
					Microphthalmia, syndromic 9 (MIM# 601186)	*STRA6* (NM_001199042.2)	c.1402 G > C p.(Ala468Pro)	AR	Hom
Family 2	Present	III-2	Developmental delay, increased serum creatine phosphokinase, family history of elder sibling with pain insensitivity	Undiagnosed	McArdle disease (MIM# 232600)	*PYGM* (NC_000011.9)	g.64519395 C > T	AR	Hom
					Congenital insensitivity to pain with anhidrosis (MIM# 256800)	*NTRK1* (NC_000001.10)	g.156834227 G > T	AR	Hom
		III-1	Developmental delay, pain insensitivity, expired at 8 months	Not examined	NA	NA	NA	NA	NA
Family 3	Present	III-1	Developmental delay, molar tooth sign and cerebellar vermis agenesis on MRI brain	Joubert syndrome	Joubert syndrome 17 (MIM# 614615)	*CPLANE1* (NM_023073.3)	c.8710 C > T p.(Arg2904Ter)	AR	Hom
		III-2	Developmental delay, growth retardation, malar rash	Cockayne syndrome	Xeroderma pigmentosum, group A (MIM# 278700).	*XPA* (NM_000380.3)	c.331 G > T p.(Glu111Ter)	AR	Hom
Family 4	Absent	II-1 II-2	Couple with previous neonatal deaths due to epidermolysis bullosa	NA	Epidermolysis bullosa dystrophica, autosomal recessive type (MIM# 226600)	*COL7A1* (NC_000003.11)	g.48605605 T > C	AR	Het
		III-3	Neuroregression and MRI brain showing leukoencephalopathy	Mitochondrial leukoencephalopathy	Mitochondrial complex I deficiency, nuclear type 7 (MIM# 615688)	*NDUFV2* (NM_021074.5)	c.548 C > T p.(Ala183Val)	AR	Hom
Family 5	Present	III-1	Neuroregression, hypertonia, pendular nystagmus, microcephaly	Undiagnosed	Microcephaly, short stature, and impaired glucose metabolism 1(MIM# 616033)	*TRMT10A* (NM_152292.5*)*	c.23dup p.(Phe9IlefsTer3)	AR	Hom
					Metachromatic leukodystrophy (MIM# 250100)	*ARSA* (NC_000022.10)	g.51064581 C > T	AR	Hom
Family 6	Present	IV-1	Ichthyosis, neuroregression, cherry red spots, hepatosplenomegaly	GM1 gangliosidosis and ichthyosis	GM2 gangliosidosis, AB variant (MIM# 272750)	*GM2A* (NC_000005.9)	g.150644873-150647042del	AR	Hom
					Autosomal recessive congenital ichthyosis 6 (MIM# 612281)	*NIPAL4* (NM_001099287.1)	c.527 C > A p.(Ala176Asp)	AR	Hom
Family 7	Present	III-1	Intellectual delay, coarse face, enzyme assay suggestive of Sanfilippo disease [Mucopolysaccharidosis type III (MPS) III]	MPS III	Mucopolysaccharidosis type IIIA (Sanfilippo A) (MIM# 252900)	*SGSH* (NM_000199.5)	c.111 T > A p.(Ser37Arg)	AR	Hom
					Spastic paraplegia 11, autosomal recessive (MIM# 604360)	*SPG11* (NM_025137.4)	c.3895 G > A p.(Glu1299Lys)	AR	Hom
		III-2	Neuroregression following a viral illness, hypotonia, normal deep tendon reflexes	Undiagnosed	Spastic paraplegia 11, autosomal recessive (MIM# 604360)	*SPG11* (NM_025137.4)	c.3895 G > A p.(Glu1299Lys)	AR	Hom
					Mitochondrial complex I deficiency, nuclear type 17 (MIM# 618239)	*NDUFAF6* (NM_152416.4)	c.[250 C > T];[620 T > C] p.[(Arg84Ter)];[(Ile207Thr)]	AR	Comp het
Family 8	Absent	III-2	Deafness, blurring of vision, generalised hypopigmentation, light coloured iris	Waardenburg syndrome	Usher syndrome, type 2 C (MIM# 605472)	*ADGRV1* (NM_032119.3)	c.1608C > G p.(Tyr536Ter)	AR	Hom
					Albinism, oculocutaneous, type IA (MIM#203100)	*TYR* (NM_000372.4)	c.575 C > A p.(Ser192Tyr)	AR	Hom
Family 9	Absent	III-3	Heterochromia iris, telecanthus, white forelock, bilateral profound to severe hearing loss and hypopigmented patches of skin on the forehead, chest and upper limbs. Father (II-2) and paternal grandmother (I-2) had heterochromia iridis. Mother (II-3) had a white forelock	Waardenburg syndrome type 1	Waardenburg syndrome type 1 (MIM# 193500)	*PAX3* (NM_181459.3)	c.166 C > T p.(Arg56Cys)	AD	Het Inherited from mother
					Waardenburg syndrome type 4 A (MIM #277580)	*EDNRB* (NM_001201397.1)	c.1047delC p.(Val350PhefsTer36)	AD	Het Inherited from father
Family 10	Absent	III-1	Telecanthus, heterochromia iridis, white forelock and bilateral hand contractures, hearing loss in both parents, paternal uncle and paternal grandmother (II-1, II-2, II-3, I-2)	Waardenburg syndrome type 3	Waardenburg syndrome type 3 (MIM# 277580)	*PAX3* (NM_181459.3)	c.829 C > T p.(Gln277Ter)	AD	Het
					Deafness, autosomal recessive 1 A (MIM# 220290)	*GJB2* (NM_004004.5)	c.71 G > A p.(Trp24Ter)	AR	Hom
Family 11	Present	IV-3	Neuroregression, myoclonic epilepsy	Myoclonic epilepsy syndrome	Epilepsy, progressive myoclonic 3, with or without intracellular inclusions (MIM# 611726)	*KCTD7* (NM_153033.5)	c.205 C > G p.Leu69Val	AR	Hom
					Autosomal dominant neutrophilic dermatosis, acute febrile (MIM #608068)	*MEFV* (NM_000243.3)	c.726 C > A p.Ser242Arg	AD	Het
Family 12	Present	IV-1	Global developmental delay, seizures, spasticity, microcephaly, cystic changes in the fronto-temporal white matter	Undiagnosed	Aicardi-Goutières syndrome 3 (MIM# 610329)	*RNASEH2C* (NM_032193.3)	c.205 C > T p.(Arg69Trp)	AR	Hom
					Neurodevelopmental disorder with microcephaly, seizures, and cortical atrophy (MIM# 617802)	*VARS1* (NM_006295.3)	c.[2086 G > C];[3695 C > A] p.[(Gly696Arg)];[(Pro1232Gln)]	AR	Comp het
Family 13	Absent	III-2	Global developmental delay, seizures, sensorineural hearing loss, microphthalmia, hypopigmented patches on forehead, abdomen and hyperpigmented patch on the right leg	Waardenburg syndrome	Rett syndrome (MIM# 312750)	*MECP2 (NM_001110792.2)*	c.538 C > T, p.Arg180Ter	XLD	De novo
					4q12 deletion	NA	NA	NA	De novo
Family 14	Absent	III-3	Developmental delay, seizures, hypotonia, microcephaly	Neurodevelopmental disorder with epilepsy	Developmental and epileptic encephalopathy 7 (MIM# 613720)	*KCNQ2 (*NM_172107.4)	c.316 T > G p.(Cys106Gly)	AD	De novo
					Becker muscular dystrophy (MIM# 300376)	290 kb deletion at cytoband Xp21.1 spanning *DMD* gene		XL	Inherited from mother

*AR* autosomal recessive, *AD* autosomal dominant, *Hom* homozygous, *Het* heterozygous, *Comp het* compound heterozygous, *XLD* X linked dominant, *XL* X linked, *NA* not applicable.

^a^The variants are reported against GRCh37/hg19 version of the human genome.

## Discussion

A systematic input of clinical information to the genome-wide tests has aided the dissection of complex phenotypes including those arising due to the presence of MGVs. We identified a cohort of 14 families retrospectively, for discussing the challenges in diagnosis and counselling in families with MGVs and MGDs. A detailed family history, deep and systematic phenotyping, follow up evaluation and clinical inputs for the analysis were complementary to the unbiased candidate prioritisation of genomic data.

In our cohort, the incidence of MGVs in families who underwent WES was 2.4%, comparable to previous reports. Individuals with MGVs have been previously discussed in the literature in terms of phenotypic presentation in affected individuals mainly as blended and distinct phenotypes. The elucidation of MGVs in a family appears to be more prudent and is likely to have a wider impact than recognition of MGVs in individuals. We observed five different consequences of MGVs i.e., (i) individuals with MGVs presenting as blended phenotypes (ii) individuals with MGVs presenting with distinct phenotypes (iii) individuals with MGVs with age-dependent penetrance (iv) individuals with MGVs with one phenotype obscured by another more predominant phenotype (v) two distinct phenotypes in different individuals in families with MGVs.

In an affected individual, MGVs can result in MGDs with either a blended or as two or more recognisable distinct phenotypes. In a blended phenotype, the individual is presumed to have a single undiagnosed condition. In our cohort, the blended phenotype was presumed in the probands in 6 out of 12 families (50%) (F5, F8, F9, F10, F12, F13). In the cohort described by Balci et al., blended phenotype was observed in 6 out of 8 families (75%) [4]. Hearing loss and hypopigmentation in the proband (III-2) in F8, mimicking a clinical diagnosis of Waardenburg syndrome, is an example of blending in of phenotypes resembling a single disorder. Interestingly, he had albinotic fundus without any evidence of retinitis pigmentosa in spite of carrying a known variant in *ADGRV1* causing Usher syndrome type 2 C. Similarly in F13, the clinical features in the proband were presumed to be caused due to Waardenburg syndrome. In F5, the proband (III-1) had microcephaly, growth retardation, regression of milestones, spasticity and subtle periventricular white matter abnormalities and was presumed to have a single genetic disease. The variants in *ARSA* and *TRMT10A* could explain these findings in her. Individuals with microcephaly, short stature, and impaired glucose metabolism 1 (MIM# 616033) due to *TRMT10A* variants are known to be at risk for early-onset diabetes, usually by the second decade. The diagnosis of multiple genetic diseases as evident in this family may provide a window of opportunity for screening and monitoring for treatable complications.

In F1 and F6, distinct phenotypes of two disorders were observed at presentation. In F1, the proband (III-1) had microphthalmia and osteogenesis imperfecta, with variants in *STRA6* and *SERPINF1* respectively. In proband IV-1 of F6, clinical features of two distinct disorders i.e., ichthyosis and a storage disorder with cherry red spots were evident at presentation. WES was carried out in view of the genetic heterogeneity of both these conditions. A large deletion in the *GM2A* gene was noticed on manual inspection of genes known to cause storage disorders with cherry-red spot, indicating the relevance of systematic phenotyping and clinical input in analysis of WES data.

MGD with age-dependent penetrance was observed in two families (F2 and F11). In F2, the proband (III-2) came to clinical attention due to family history, parental concerns and biochemical findings of high CPK at 5 months of age. At that time, the variant in *PYGM* was reported, which could explain the elevated CPK. When she was re-evaluated at 1 year 5 months, she had evidence of self-mutilation. Reanalysis of WES data revealed a variant, c.287 + 7 G > T in intron 2 of *NTRK1* [NC_000001.10 (NM_002529.3)] confirming the diagnosis of congenital insensitivity to pain with anhidrosis (MIM# 256800) in III-2. This variant was at a non-canonical splice site and hence was not considered in the initial analysis in the absence of clinical findings. In F11, the proband IV-3 had early-onset epilepsy with homozygous variants in *KCTD7*. The second heterozygous variant observed in *MEFV* was identified in the WES data analysis as it was a previously reported variant known to cause autosomal dominant neutrophilic dermatosis, acute febrile (MIM# 608068). This condition has childhood-onset and no overt clinical findings were observed at the time of examination. Unfortunately, the family was lost to follow up and we were unable to re-evaluate later.

Five families (F7, F9, F10, F12, F14), represent a scenario where one Mendelian disorder resulted in a predominant phenotype, obscuring the second disorder. In F7, the findings were largely attributable to mitochondrial complex I deficiency in III-1 and Sanfillipo A disease in III-2. The phenotype of spastic paraplegia, which usually manifests in the latter half of the first decade or second decade of life, could not be confirmed in both these siblings who already had a genetic disorder with a more severe phenotype at an earlier age. In F9, the proband (III-3) had the phenotype of *PAX3*-related Waardenburg syndrome type 1 (WS) (congenital hearing loss, heterochromia iridis, telecanthus and hypopigmentation of hair and skin). In spite of having another variant in *EDNRB*, Hirschsprung disease was absent. In this family, both the maternal and paternal side of the family had features of WS, hence prompting us to look for variants inherited from both the father as well as the mother. In F10, the phenotype of hearing loss in III-1 was overshadowed by the presence of clinical findings of PAX3-related WS. However, the variant causing WS, observed in the proband was not inherited from the mother, prompting us to look for other causative variants for her hearing loss phenotype. In F12, the proband (IV-1) had characteristic phenotypic findings of Aicardi-Goutières syndrome 3 (AGS). Two missense variants in *VARS1* were observed in the compound heterozygous state. The more severe neurodevelopmental phenotype and the MRI imaging findings of AGS made it challenging to carry out the clinical correlation of the variants in *VARS1*. However, dysmorphism in the proband was strikingly similar to the previously reported individuals with pathogenic variants in *VARS1*. In such families with definitely overlapping phenotypes, even with “reverse phenotyping”, where genomic data drove the search for phenotypes, it would be difficult to differentiate the extent of contribution of a particular genotype to the phenotype [14]. These scenarios posed several challenges in return for genomic results. The counselling in these families involved delivering the complexity of contribution of each of the reported variants for the phenotype in question and was further complicated by the phenomenon of variable expression, non-penetrance and often variants of uncertain significance.

In F3, F4 and F7, MGVs resulted in more than one disorder in the family but not MGDs in individuals. In F3, the parents were carriers of the variants in *CPLANE1* and *XPA*. Their first and second offspring (III-1 and III-2) had Joubert syndrome 17 and xeroderma pigmentosum, group A, respectively. Genetic testing in this family was carried out after the birth of the second child, thus delaying the diagnosis of MGVs in the family. In F4 and F7, one genetic disease was identified in the first affected child. Prenatal diagnosis was done in subsequent pregnancies for the same condition. However, in both these families, the next offspring had a second genetic disorder as parents were carriers for more than one disorder. The couple in F4 underwent carrier screening by WES to look for variants relevant to the phenotype of their deceased children with epidermolysis bullosa. The screening in these individuals was targeted and hence other variants, largely the variant of uncertain significance including the novel missense variants, in *NDUFV2*, which caused mitochondrial complex I deficiency, nuclear type 7 (MIM# 615688) in their second offspring, were not looked into [15]. Hence, we recommend that the limitation of carrier screening by NGS techniques should be conveyed to the families as part of pre-test counselling.

MGDs are observed more commonly in populations with a high prevalence of consanguinity. The overall rate of consanguinity is not available for families seen at our centre during the period of study. However, of the 850 families who underwent WES, 331 families were consanguineous (38.9%) and 410 families (48.2%) were non-consanguineous. Data on consanguinity was not available for 109 families. In our cohort, consanguinity was seen in 8 of the 14 families (57.1%). The rate of multiple genetic diagnosis in our cohort in consanguineous families is 2.4% (8/331) and non-consanguineous families are 1.4% (6/410). This is similar to the previously reported studies, which reported a higher rate of multiple genetic diagnoses in consanguineous populations [3, 9]. In our cohort, in one of the consanguineous families (F11), we identified a de novo variant in addition to an autosomal recessive disorder. Homozygous variants were identified in two non-consanguineous families, F4 and F10. This could be because of hidden consanguinity, endogamy or inbreeding which are prevalent in India.

The most common inheritance pattern observed in our cohort was the presence of two autosomal recessive diseases as observed previously [4]. Eight families in our cohort had two autosomal recessive disorders (Family 1, 2, 3, 4, 5, 6, 8, 12). Consequently, most pathogenic variants were seen in genes causing autosomal recessive disorders. In the study by Posey et al., the most common variants were identified in autosomal dominant conditions and the most common pattern observed was pathogenic variants in two autosomal dominant disorders [3].

The presence of MGVs in a family has several implications in genetic counselling. However, the most significant implication is on the risk of recurrence and prenatal diagnosis. Punnett square to assess the risk of recurrences in different scenarios is provided in Supplementary Fig. 2. A careful evaluation of risk figures is warranted in these families. When two autosomal recessive disorders which segregate independently are present in a family, the risk of having a child with neither of the two diseases will be 56% (Supplementary Fig. 2A). However, as observed in proband in F6 (IV-1) the genes *NIPAL4* and *GM2A* are in very close proximity to each other on chromosome 5 (*NIPAL4*: chr5:156,886,027-156,902,730 and *GM2A*: chr5: 150,631,746-150,651,000). The chances of recombination and thus independent segregation will be low and these genes will be most likely inherited as a single unit. Thus, the chance of having an affected child for this family is likely to be 25%, similar to that of a single recessive disease. In a family with three recessive disorders, the chance of having a child without any of the three autosomal recessive disorders will be 42% (F7 with three different genetic diagnoses) (Supplementary Fig. 2C). The chance of having any one of these autosomal recessive disorders is 42%. The chance of an offspring having any two autosomal recessive disorders will be 14%. Two autosomal dominant conditions were identified in F9. The chance of this family having an unaffected child without the two dominant conditions would be 25% (Supplementary Fig. 2B). The risk of recurrence will depend on whether the dominant condition is inherited from a parent or de novo. The proband in F10 had one dominant and one recessive condition. In this family, the testing in the father could not be completed and hence recurrence risk could not be accurately predicted. The proband (IV-3) in F11 had an autosomal recessive disorder and an autosomal dominant disorder caused due to a de novo variant. His parents were carriers for an autosomal recessive disorder. In such a family, the chance of autosomal recessive disorder in offspring would be 25% and the chance of a de novo variant occurring again would be less than 1%.

## Conclusion

MGVs and MGDs present a complex and challenging scenario for both the clinic as well as the laboratories performing genomic testing. The study highlights the impact of systematic phenotyping through inputs of medical geneticists on the analysis and thus recognition of MGVs in the genomic data. The presence of MGVs may often blur the boundary of primary and secondary findings in individuals and families undergoing genomic testing. However, a careful dissection of the phenotype and a concurrent unbiased evaluation of the genomic information aids accurate definitive diagnoses, genetic counselling and reproductive choice in these families.

## Supplementary information

Supplemental material
