# Supplementary material for: Multilocus disease-causing genomic variations for Mendelian disorders: role of systematic phenotyping and implications on genetic counselling
Source: Eur J Hum Genet. 2021 Jul 19;29(12):1774–80. doi: 10.1038/s41431-021-00933-7 (PMC8633282; doi:10.1038/s41431-021-00933-7)
Supplement: Supplementary file 1 — Supplemental material [file 41431_2021_933_MOESM1_ESM.docx]

**Supplementary Table 1: Technical details of whole exome sequencing (WES)**

| **Family ID** | **WES approach** | **Capture kit** | **Mean depth** | **Coverage (%)** | |
| --- | --- | --- | --- | --- | --- |
|  |  |  |  | **≥10x** | **≥20x** |
| Family 1 | Solo* | Illumina Nextera Rapid Capture Exomes | 108.16 | 98 | 95 |
| Family 2 | Solo | Illumina Nextera Rapid Capture Exomes | 71.59 | 95 | 89 |
| Family 3 | Duo^#^ | Agilent SureSelect CREv2 | 112.13 | 99 | 96 |
| Family 4 | Duo | Illumina Nextera Rapid Capture Exomes | 113.10 | 97 | 93 |
|  | Solo | Agilent SureSelect CREv2 | 85.80 | 98 | 94 |
| Family 5 | Solo | Agilent SureSelect CREv2 | 98.49 | 98 | 94 |
| Family 6 | Solo | Illumina Nextera Rapid Capture Exomes | 101.54 | 96 | 92 |
| Family 7 | Solo | Illumina Nextera Rapid Capture Exomes | 82.79 | 95 | 89 |
| Family 8 | Solo | Illumina Nextera Rapid Capture Exomes | 99.33 | 96 | 90 |
| Family 9 | Duo | Illumina Nextera Rapid Capture Exomes | 88.74 | 95 | 88 |
| Family 11 | Solo | Illumina Nextera Rapid Capture Exomes | 75.07 | 96 | 89 |
| Family 12 | Solo | Agilent SureSelect CREv2 | 87.21 | 98 | 94 |
| Family 13 | Solo | Agilent SureSelect CREv3 | 76.28 | 71.1 | 68.4 |
| Family 14 | Trio^$^ | Agilent SureSelect CREv2 | 138.66 | 99.5 | 98.1 |

*Solo: Sequencing of the proband

#Duo: Sequencing of another member in addition to the proband

$Trio: Sequencing of the proband, his/her mother and father

**Supplementary Table 2: Phenotype and genotype of families**

| **Family ID** | **Subject ID** | **Conditions identified** | **Inheritance pattern** | **Gene  (Transcript)** | **Variants*** | **Zygosity** | **Known**  **/novel** | ***In silico tools prediction*** | **Genomic coordinates** | **Allele counts** | | | | **ACMG variant classification**  (criteria) |
| --- | --- | --- | --- | --- | --- | --- | --- | --- | --- | --- | --- | --- | --- | --- |
|  |  |  |  |  |  |  |  |  |  | **gnomAD counts#** | | **In house counts** | |  |
|  |  |  |  |  |  |  |  |  |  | **Number of homozygotes** | **Number of heterozygotes** | **Number of homozygotes** | **Number of heterozygotes** |  |
| **Family 1** | **III-1** | Osteogenesis imperfecta type VI (MIM# 613982) | AR | *SERPINF1 (NM_ 002615.7)* | c.838_839delCT p.(Leu280Glufs*20) | Hom | Novel | - | 17-1679877_1679878delCT | 0 | 0 | 0 | 0 | Pathogenic  (PVS1, PM2, PP3) |
|  |  | Microphthalmia, syndromic 9 (MIM# 601186) | AR | *STRA6 (NM_001199042.2)* | c.1402G>C p.(Ala468Pro) | Hom | Novel | MutationTaster, REVEL and SIFT | 15-74476212-C-G | 0 | 0 | 0 | 0 | Likely pathogenic  (PM1, PM2, PP3, PP4) |
| **Family 2** | **III-2** | McArdle disease (MIM# 232600) | AR | *PYGM  (NC_000011.9)* | g.64519395C>T | Hom | Known | CADD score:19.3 | 11-64519395-C-T | 0 | 7 | 0 | 0 | Pathogenic  (PVS1, PM2, PP3) |
|  |  | Congenital insensitivity to pain with anhydrosis (MIM# 256800) | AR | *NTRK1  (NC_000001.10)* | g.156834227G>T | Hom | Novel | Splice AI and HSF: New donor site activation | chr1-156834227-G-T | 0 | 0 | 0 | 0 | VOUS (PM2, PP3) |
| **Family 3** | **III-1** | Joubert syndrome 17 (MIM# 614615) | AR | *CPLANE1  (NM_023073.3)* | c.8710C>T p.(Arg2904Ter) | Hom | Known | FATHMM-MKL and MutationTaster | chr5-37125432-G-A | 0 | 9 | 0 | 1 | Pathogenic  (PVS1, PM2, PP3) |
|  | **III-2** | Xeroderma Pigmentosum, group A (MIM# 278700). | AR | *XPA  (NM_000380.3)* | c.331G>T p.(Glu111Ter) | Hom | Known | FATHMM-MKL and MutationTaster | chr9-100451874-C-A | 0 | 4 | 0 | 1 | Pathogenic  (PVS1, PM2, PP3) |
| **Family 4** | **II-1 II-2** | Epidermolysis bullosa dystrophica, autosomal recessive type (MIM# 226600) | AR | *COL7A1m(NC_000003*  *.11)* | g.48605605T>C | Het | Novel | HSF and NetGen2: Alteration of acceptor splice site | chr3-48605605-T-C | 0 | 0 | 0 | 3 | Pathogenic  (PVS1, PM2, PP3) |
|  | **III-3** | Mitochondrial complex I deficiency, nuclear type 7 (MIM# 615688) | AR | *NDUFV2 (NM_021074.5)* | c.548C>T p.(Ala183Val) | Hom | Novel | Mutation Taster, SIFT, REVEL and M-CAP | chr18-9124950-C-T | 0 | 1 | 1 | 0 | VOUS  (PM2, PP3, PP4) |
| **Family 5** | **III-1** | Microcephaly, short stature, and impaired glucose metabolism 1 (MIM# 616033) | AR | *TRMT10A  (NM_152292.5)* | c.23dup p.(Phe9IlefsTer3) | Hom | Novel | - | chr4-100480472-T-TG | 0 | 0 | 0 | 0 | Pathogenic  (PVS1, PM2, PP3) |
|  |  | Metachromatic leukodystrophy (MIM# 250100) | AR | *ARSA  (NC_000022.10)* | g.51064581C>T | Hom | Known | - | chr22-51064581-C-T | 0 | 2 | 0 | 0 | Pathogenic  (PVS1, PM2, PP3, PP5) |
| **Family 6** | **IV-1** | GM2 gangliosidosis, AB variant (MIM# 272750) | AR | *GM2A  (NC_000005.9)* | g.150644873-150647042del | Hom | Novel |  | chr5:150,644,873-150,647,042 (2170bp) | 0 | 0 | 0 | 0 | Pathogenic  (PVS1, PM2, PP3) |
|  |  | Autosomal recessive congenital ichthyosis 6 (MIM# 612281) | AR | *NIPAL4  (NM_001099287.1)* | c.527C>A p.(Ala176Asp) | Hom | Known | MutationTaster | chr5-156895736-C-A | 0 | 184 | 0 | 0 | Pathogenic  (PM1, PM2, PP2, PP3, PP5) |
| **Family 7** | **III-1** | Mucopolysaccharidosis type IIIA (Sanfilippo A) (MIM# 252900) | AR | *SGSH  (NM_000199.5)* | c.111T>A p.(Ser37Arg) | Hom | Novel | MutationTaster | chr17-78190969-A-T | 0 | 1 | 0 | 1 | VOUS  (PM1, PM2) |
|  |  | Spastic paraplegia 11, autosomal recessive (MIM# 604360) | AR | *SPG11  (NM_025137.4)* | c.3895G>A p.(Glu1299Lys) | Hom | Novel | MutationTaster | chr15-44890569-C-T | 0 | 10 | 0 | 1 | Likely pathogenic  (PM2, PP1, PP3, PP4) |
|  | **III-2** | Spastic paraplegia 11, autosomal recessive (MIM# 604360) | AR | *SPG11  (NM_025137.4)* | c.3895G>A p.(Glu1299Lys) | Hom | Novel | MutationTaster | chr15-44890569-C-T | 0 | 10 | 0 | 1 | Likely pathogenic  (PM2, PP1, PP3, PP4) |
|  |  | Mitochondrial complex I deficiency, nuclear type 17 (MIM# 618239) | AR | *NDUFAF6  (NM_152416.4)* | c.[250C>T];[620T>C] p.[(Arg84Ter)];[(Ile207Thr)] | Comp het | Novel | MutationTaster | chr8-96044275-C-T chr8-96059261-T-C | 0 0 | 3 1 | 0 0 | 0 0 | Pathogenic  (PVS1, PM2, PP3)  Likely pathogenic  (PM2, PM3, PP3, PP4) |
| **Family 8** | **III-2** | Usher syndrome, type 2C (MIM# 605472) | AR | *ADGRV1  (NM_032119.4)* | c.1608C>G p.(Tyr536Ter) | Hom | Known | MutationTaster CADD:35 | chr5-89925125-C-G | 0 | 0 | 0 | 1 | Pathogenic  (PVS1, PM2, PP3, PP5) |
|  |  | Albinism, oculocutaneous, type IA (MIM# 203100) | AR | *TYR  (NM_000372.4)* | c.575C>A p.(Ser192Tyr) | Hom | Known | MutationTaster | chr11-88911696-C-A | 11859 | 47026 | 8 | 113 | VOUS  (BA1, BP6, PM1, PS3) |
| **Family 9** | **III-3** | Waardenburg syndrome type 1 (MIM# 193500) | AD | *PAX3  (NM_181459.3)* | c.166C>T  p.(Arg56Cys) | Het | Novel | MutationTaster | chr2-223161852-G-A | 0 | 0 | 0 | 1 | Pathogenic  (PS3, PM1, PM2, PM5, PP2, PP3) |
|  |  | Waardenburg syndrome type 4A (MIM# 277580) | AD | *EDNRB  (NM_001201397.1)* | c.1047delC p.(Val350PhefsTer36) | Het | Novel | MutationTaster | chr13-78477315-CG-C | 0 | 9 | 0 | 1 | Pathogenic  (PVS1, PM2, PP3) |
| **Family 10** | **III-1** | Waardenburg syndrome type 3 (MIM# 277580) | AD | *PAX3  (NM_181459.3)* | c.829C>T p.(Gln277Ter) | Het | Novel | MutationTaster | chr2-223086070-G-A | 0 | 0 | 0 | 0 | Pathogenic  (PVS1, PS3, PM2, PP3) |
|  |  | Deafness, autosomal recessive 1A (MIM# 220290) | AR | *GJB2  (NM_004004.5)* | c.71G>A  p.(Trp24Ter) | Hom | Known | - | chr13-20763650-C-T | 1 | 145 | 0 | 30 | Pathogenic  (PVS1, PM2, PP3) |
| **Family 11** | **IV-3** | Epilepsy, progressive myoclonic 3, with or without intracellular inclusions (MIM# 611726) | AR | *KCTD7  (NM_153033.5)* | c.205C>G p.(Leu69Val) | Hom | Novel | MutationTaster SFT, Polyphen | chr7-66098322-C-G | 0 | 0 | 0 | 1 | Likely pathogenic (PM1, PM2, PP2, PP3) |
|  |  | Autosomal dominant neutrophilic dermatosis, acute febrile (MIM# 608068) | AD | *MEFV  (NM_000243.3)* | c.726C>A p.(Ser242Arg) | Het | Known | MutationTaster, SIFT, M-CAP | chr16-3304342-G-T | 0 | 0 | 0 | 2 | Likely pathogenic  (PS3, PM2, PM6) |
| **Family 12** | **IV-1** | Aicardi-Goutières syndrome 3  (MIM# 610329) | AR | *RNASEH2C*  *(NM_032193.3)* | c.205C>T  p.(Arg69Trp) | Hom | Known | SIFT  PROVEAN  FATHMM  M-CAP  MetaSVM  CADD-26.6 | Chr11-65487856-G-A | 0 | 23 | 2 | 0 | Pathogenic (PS3, PM2, PP3, PP5) |
|  |  | Neurodevelopmental disorder with microcephaly, seizures, and cortical atrophy (MIM# 617802) | AR | *VARS1*  *(NM_006295.3)* | c.[2086G>C];[3695C>A]  p.[(Gly696Arg)];[(Pro1232Gln)] | Comp het | Novel | SIFT  LR  MutationTaster  PROVEAN  fathmm-MKL  CADD-26.6 | Chr6-31750126-C-G  Chr6-31746775-G-T | 0  0 | 21  0 | 0  0 | 1  0 | VOUS (PM2, PP3)  VOUS (PM2, PP3) |
| **Family**  **13** |  | Rett syndrome (MIM# 312750) | XLD | *MECP2*  *(NM_001110792.2)* | c.538C>T p.(Arg180Ter) | De novo | Known | MutationTaster, MutPred, FATHMM-MKL, CADD: 38 | Chr X-153296777-C-T | 0 | 0 | 0 | 1 | Pathogenic  (PVS1, PM2, PP3) |
|  |  | 4q12 deletion | AD | arr[GRCh38] 4q12q21  (4:51891814-76009719) x 1 |  | Unknown | Novel |  | Chr4-51891814-76009719 | NA | NA | NA | NA | NA |
| **Family 14** | **III-3** | Developmental and epileptic encephalopathy 7 (MIM# 613720) | AD | *KCNQ2 (NM_172107.4)* | c.316T>G p.(Cys106Gly) | De novo | Novel | SIFT, PolyPhen, MutationTaster, M-CAP, CADD: 21 | Chr20-62078171-A-C | 0 | 0 | 0 | 0 | Likely pathogenic (PS2, PM2, PP2, PP3, PP4) |
|  |  | Becker muscular dystrophy (MIM# 300376) | XL | 290kb deletion at cytoband Xp21.1 spanning *DMD* gene |  | Inherited from mother |  |  |  | NA | NA | NA | NA | NA |

*The variants are reported against GRCh37/hg19 version of human genome, ^#^gnomAD v.2.1.1 and inhouse exome data of 1196 individuals, ACMG- American College of Medical genetics and Genomics, VOUS- variant of uncertain significance, AR – autosomal recessive, AD – autosomal dominant, Hom- Homozygous, Het- heterozygous, Comp het – Compound heterozygous, XLD: X linked dominant, XL: X linked

**Whole exome sequencing data processing and variant annotation**

The generated raw reads from exome sequencing were subjected to initial quality assessment using FastQC toolkit (Andrews, 2010) followed by coverage analysis by employing GATK v3.6 ‘Depth of Coverage’ (Van der Auwera et al., 2013) . The raw reads were aligned to the human reference genome (GRCh37) using BWA-MEM (v0.7.15) (Li, 2013) and the generated alignment were subjected to series of post processing steps following the GATK best practices for germline SNV and INDEL discovery from exome sequences. Picard (v.2.5.0) (Picard) was used to sort and index the resulting alignment followed by realignment around known Indels and SNVs using GATK ‘Realigner Target Creator’ and ‘Indel Realigner’. Base quality score recalibration was performed using GATK ‘Base Recalibrator’ and then the genomic VCF (gvcf) file was generated using GATK ‘Haplotype Caller’ with the appropriate exome capture kit bed file. Joint genotyping was performed for the entire cohort, followed by GATK variant quality recalibration (VQSR) and left normalization using BCFTOOLS (v1.3.1) (Li, 2011) to generate a multi-sample VCF file. Allele state (counts of heterozygotes and homozygotes), were derived using customized Perl scripts. GATK ‘Select Variants’ was used to extract the variants called per sample based on the respective sample ID to create a single sample variant call format (VCF) file. The single sample VCF file was then annotated against ‘RefGene’, ‘gnomad_exome’, ‘gnomad_genome’, ‘snp138’, ‘clinvar_20190305’, ‘exac03’ ‘dbscsnv11’ ‘dbnsfp35c’ ‘mcap’ ‘intervar_20180118’, ‘revel’, ‘clinpred’ and ‘avsnp150’ using ANNOVAR (Wang, Li, &amp; Hakonarson, 2010) . Counts of heterozygotes and homozygotes resulted based on the joint genotyping of the entire cohort along with allele counts and number of homozygotes observed in gnomAD were integrated using ANNOVAR ‘generic dbfile’. Downstream to ANNOVAR annotations, in-house Perl scripts were used to integrate disease phenotypes catalogued in OMIM (Online Mendelian Inheritance in) and HPO terms.

**Technical details of chromosomal microarray**

1. **Proband in Family 13:**

Affymetrix CytoScan^TM^ 750K Array (Santa Clara, California, United States)

Interpretation of CMA data was carried out using Chromosome Analysis Suite (ChAS) v4.1 software. CNV loss of >50 Kb and gain of >200 Kb in all regions are reported. However, smaller CNVs are reported if present in well described CNV regions or associated with regulatory regions of genes. Benign copy number variants and LOH region less than 8 Mb will not be disclosed. Variants in cancer predisposition genes or carrier status of any variant known to cause autosomal recessive disease or X linked diseases are not reported. We do not disclose CNVs unrelated to reasons for referral (secondary findings). CNVs are reported according to the International System for Human Cytogenomic Nomenclature (ISCN) and American College of Medical Genetics and Genomics (ACMG) guidelines.

**2. Proband in Family 14:**

Illumina HumanCytoSNP-12 BeadChip (San Diego, California, United States)

Interpretation was carried out using KaryoStudio v1.4 software. Gain or loss of more than or equal to 200Kb in all regions are reported. However, smaller CNVs are reported if present in well described CNV regions or associated with regulatory regions of genes. Benign copy number variants and LOH region less than 8 Mb will not be disclosed. Variants in cancer predisposition genes or carrier status of any variant known to cause autosomal recessive disease or X linked diseases are not reported. We do not disclose CNVs unrelated to reasons for referral (secondary findings). CNVs are reported according to the International System for Human Cytogenetic Nomenclature and ACMG guidelines.

**Clinical details of individual families with multiple disease-causing genomic variants**

**Family 1**

One-year-eight months old female (III-1), one of a twin, born preterm, to third degree consanguineous parents, was evaluated for developmental delay, unilateral microphthalmia and history of repeated fractures (**Supplementary Figure 1a**). At the time of examination, she could sit with support and follow simple commands. She had three fractures by the age of one-year-eight-months. On examination, her weight was 6.6 kg (-3.2 SD), length was 71 cm (-3.5 SD), and occipito-frontal circumference was 44 cm (normal). She had left microphthalmia, mild frontal prominence and bowing of both thighs. There was no joint laxity. Radiographs showed generalised osteopenia and fracture of left femur (**Supplementary Figure 1b**). She was clinically diagnosed to have distinct phenotypes of osteogenesis imperfecta and microphthalmia. Whole exome sequencing (WES) was done for the proband and a novel homozygous variant c.838_839delCT p.(Leu280Glufs*20) in exon 7 of *SERPINF1* (NM_ 002615.7), causing osteogenesis imperfecta Type VI (MIM# 613982) and a novel homozygous missense variant c.1402G>C p.(Ala468Pro) in exon 14 of *STRA6* (NM_001199042.2) *,* causing microphthalmia, syndromic 9 (MIM# 601186) were identified. Both the variants were found in heterozygous states in her parents.

**Family 2**

A 5-months-old female (III-2), second born to third degree consanguineous parents, was evaluated for delayed development. There was family history of an elder male sibling who had global developmental delay and recurrent febrile illness since 3 months of age. He was also noticed to have less sensitivity to pain during immunisation and had multiple ulcers on hands. He expired at 8 months of age following febrile illness. On examination, she did not have any facial dysmorphism. She did not have any bite marks or non-healing wounds. Her length was 63cm (normal) and head circumference was 37.5cm (-3.25 SD). She had generalised hypotonia and normal deep tendon reflexes. Her fundus and hearing evaluation were normal. Serum creatine phosphokinase (CPK) was 2356 U/L (10-80 U/L). Magnetic Resonance Imaging (MRI) of the brain was normal except for mild hypoplasia of corpus callosum. At the time of examination, the proband did not have features suggestive of a disease similar to her elder sibling. A known canonical splice site variant, g.64519395C>T (NM_005609.2:c.1768+1G>A) in intron 14 of *PYGM* (NC_000011.9), known to cause McArdle disease (MIM# 232600) (1), was identified in her. This variant could explain the elevated CPK in her. She was re-evaluated at 1 year 2 months and was noted to have multiple bites on her tongue and fingers. Parents also felt that she had poor sensitivity to pain and recurrent febrile illness similar to the elder sibling. Reanalysis of WES data showed another novel homozygous splicing variant, g.156834227G>T (NM_002529.3:c.287+7G>T) in intron 2 of *NTRK1* (NC_000001 .10), suggestive of congenital insensitivity to pain with anhidrosis (MIM# 256800) in her. This could explain the developmental delay, recurrent febrile episodes and self-mutilation. Parents were found to be heterozygous carriers for both the autosomal recessive conditions.

**Family 3**

One-year-old girl (III-2), second born to first cousin parents, had developmental delay and growth retardation. She was born at term after an uneventful pregnancy by normal vaginal delivery. Her birth weight was 3.220 kg (normal). She attained neck holding at six months and started sitting independently at twelve months. Speech was present only in the form of cooing and babbling. On examination, her length was 76 cm (normal) and occipito-frontal circumference was 43 cm (normal). She had a photosensitive butterfly rash on her face (**Supplementary Figure 1d**). There was intermittent spasticity of limbs. MRI of the brain done at 1 year 3 months-of age was normal. In view of developmental delay, growth retardation and butterfly rash, she was clinically diagnosed with Cockayne syndrome. Her six-years-old female sibling (III-1) was previously evaluated for mild developmental delay. She was born at term by normal vaginal delivery. Her birth weight was 2.880 kg (normal). Perinatal period was uneventful. She attained neck holding at eight months, started sitting independently at nine months and began to walk independently at 2 years. She started speaking bisyllables at 2 years 6 months of age and could speak full sentences by 6 years of age. On examination, her weight was 22 kg (normal), height was 112 cm (normal) and occipito-frontal circumference was 54.5 cm (+2.3 SD). She had dolicocephaly, mild midface hypoplasia and strabismus. MRI of the brain done at 1 year of age showed molar tooth sign, due to cerebellar vermis dysgenesis, suggestive of Joubert syndrome (**Supplementary Figure 1c).** ES was done for the siblings. In the elder sibling, a known homozygous stop-gain variant, c.8710C>T p.(Arg2904Ter) in *CPLANE1* (NM_023073.3) causing Joubert syndrome 17 (MIM# 614615) was identified (2). Her younger sibling was a carrier for the same variant. In the younger sibling, a known homozygous stopgain variant, c.331G>T p.(Glu111Ter) in *XPA* (NM_000380.3) was identified, confirming the diagnosis of Xeroderma Pigmentosum, group A (MIM# 278700) (3). Her elder sister had the same variant in heterozygous state. Parents were heterozygous carriers for both the variants.

**Family 4**

A non-consanguineously married couple (II-1, II-2) sought counselling for prenatal diagnosis in view of two neonatal deaths clinically diagnosed as epidermolysis bullosa. Genetic evaluation was not done for the neonates. WES of the couple showed a novel canonical splice site variant, g.48605605T>C (NM_000094.4:c.7795-2G>A in *COL7A1* (NC_000003.11) in heterozygous state in both of them. Biallelic variants in *COL7A1* are known to cause epidermolysis bullosa dystrophica, autosomal recessive type (MIM# 226600). Prenatal diagnosis by chorionic villus sampling and targeted mutation analysis of fetal DNA was done and the fetus was found to be a heterozygous carrier for the same variant. This baby (III-3) was brought at 5 months of age with a history of neuroregression and MRI brain showing confluent white matter changes suggestive of leukoencephalopathy. WES of this baby identified a novel variant, c.548C>T p.(Ala183Val) in *NDUFV2* (NM_021074.5)*,* confirming the diagnosis of mitochondrial complex I deficiency, nuclear type 7 (MIM# 618229). The couple was heterozygous carrier of this variant as well.

**Family 5**

A 26-months-old female (III-1) first born to a third-degree consanguineous couple presented with complaints of neuroregression since 18 months of age. She was born at term with a birth weight of 2.5 kg (normal). She had normal development in all domains till 18 months of age. She could walk independently and speak 2-3 meaningful words. She lost all motor and language milestones over the next 8 months. On examination, her height was 73 cm (-4 SD) and her head circumference was 42 cm (-6 SD). She had horizontal pendular nystagmus and microcephaly, hypertonia in all limbs with brisk deep tendon reflexes and extensor plantar response. WES revealed two homozygous variants, a novel frameshift insertion variant, c.23dup p.(Phe9IlefsTer3) in *TRMT10A* (NM_152292.5) known to cause microcephaly, short stature, and impaired glucose metabolism 1(MIM# 616033) and a known splice site variant, c.979+1G>A in *ARSA* (NM_000487.6) (4) causing metachromatic leukodystrophy (MIM# 250100). Sanger sequencing confirmed the presence of these two variants in a homozygous state in the proband and in a heterozygous state in her parents.

**Family 6**

A one-year-seven-months old first-born female (IV-1) to a second degree consanguineous couple had ichthyosis, neuroregression and seizures (**Supplementary Figure 1e**). She was born at term after an uneventful pregnancy by normal vaginal delivery. She did not cry immediately after birth. Her birth weight was 2.7 kg (normal). She had an imperforate urethral opening, for which surgical repair was done at 48 hours of life. She attained neck holding at five months, started sitting with support at eight months and began to walk with support at twelve months. She started speaking bisyllables at twelve months of age. She started regressing after 13 months of age and lost all her attained milestones. She had unprovoked seizures starting at one-year-three-months of age for which she was started on levetiracetam. On examination, her weight was 7.5 kg (normal), total length was 75 cm (normal), and occipito-frontal circumference was 43 cm (normal). She had generalized ichthyosis, hepatosplenomegaly, and variable tone with exaggerated deep tendon reflexes. MRI brain done at 17 months revealed areas of gliosis involving centrum semiovale, corona radiata and periventricular white matter changes suggestive of mild to moderate chronic ischemic changes. Ophthalmology evaluation revealed the presence of bilateral cherry red spots. Electroencephalography (EEG) was normal. A clinical diagnosis of GM2 gangliosidosis and congenital ichthyosis was made. ES done for the proband did not show any clinically significant variants in *HEXA* and *HEXB*. Hence, manual inspection of genes known to cause neuroregression with cherry spots was carried out on Integrative Genomics Viewer (IGV). IGV showed a complete absence of reads of exon 3 and exon 4 of *GM2A*. Gap-PCR followed by Sanger sequencing confirmed a novel homozygous variant, g.150644873-150647042del (NM_000405.5:c.244-1419_*30del) in *GM2A* [NC_000005.9) encompassing partial intron 2, exon 3, intron 3, exon 4 and 3’UTR. This confirmed the diagnosis of GM2-gangliosidosis, AB variant (MIM# 272750) in the proband. Another known homozygous variant, c.527C>A p.(Ala176Asp) in exon 4 of *NIPAL4* (NM_001099287.1) was identified, confirming the diagnosis of autosomal recessive congenital ichthyosis 6 (MIM# 612281) (5). Parents were found to be carriers of both the variants.

**Family 7**

A six-years-five-months old female (III-2), second child of a third degree consanguineous couple presented with neuroregression and history of mucopolysaccharidosis type IIIA in elder male sib (III-1). She was born at term with a birth weight of 2.3 kg (-2 SD). Her mother had undergone prenatal diagnosis by amniocentesis and enzyme assay for Sanfilippo A disease, which rendered normal results. She had normal development until one-year-four-months. Following a lower respiratory tract infection, she developed neck stiffness followed by neck floppiness and stooping of back while sitting. There was no regression of milestones in other domains. On examination, she had bilateral low set ears, telecanthus and epicanthal folds. She had generalized hypotonia and normal deep tendon reflexes. She did not have hepatosplenomegaly. Fundus evaluation was normal. MRI brain done at five years of age revealed bilateral hyperintense lesions in both putamen with liquefaction and parasagittal and precentral gyri symmetrically in bilateral frontal lobes (**Supplementary figure 1f)**. III-1 was diagnosed to have mucopolysaccharidosis type IIIA (MIM# 252900) by enzyme assay. WES done for III-2 identified a novel homozygous missense variant c.3895G>A p.(Glu1299Lys) in *SPG11* (NM_025137.4) causing Spastic paraplegia 11, autosomal recessive (MIM# 604360). This variant was seen in heterozygous state in parents and homozygous state in III-1. Two novel compound heterozygous variants, c.[250C>T];[620T>C] p.[(Arg84Ter)];[(Ile207Thr)] in *NDUFAF6* (NM_152416.4), causing mitochondrial complex I deficiency, nuclear type 17 (MIM# 618239) were also identified in III-2. The variant c.250C>T p.(Arg84Ter) was seen in heterozygous state in the mother and the variant c.620T>Cp.(Ile207Thr) was seen in heterozygous state in the father. WES also identified a novel heterozygous missense variant, c.111T>A p.(Ser37Arg) in *SGSH* (NM_000199.5) in III-2. This variant was seen in homozygous state in III-1 confirming the diagnosis of mucopolysaccharidosis type IIIA in him. The same variant was seen in heterozygous state in parents. The clinical and radiological features in III-2 represented a blended phenotype of spastic paraplegia 11 and mitochondrial complex I deficiency, nuclear type 17. Her elder sibling, III-1 had mucopolysaccharidosis type IIIA (Sanfillipo A) and spastic paraplegia 11, autosomal recessive.

**Family 8**

A 19-years-old male (III-2), second born of non-consanguineous parents, was evaluated for childhood deafness and sudden blurring of vision**.** He had generalised hypopigmentation and light-coloured iris. Eye evaluation showed albinotic fundus. A clinical diagnosis of Waardenburg syndrome was made and WES was performed. No pathogenic variants in genes causing Waardenburg syndrome were identified. A known variant, c.1608C>G p.(Tyr536Ter) in *ADGRV1* (NM_032119.3) in homozygous state, known to cause Usher syndrome, type 2C (MIM# 605472) was identified. Another homozygous missense variant c.575C>A p.Ser192Tyr in *TYR* (NM_000372.4), known to cause albinism, oculocutaneous, type IA (MIM# 203100) was identified. Though this variant was reported as a polymorphism in Caucasian population with minor allele frequency of 0.2545 in gnomAD, this variant was underrepresented in Indian Genome Variant Consortium (6). This variant could explain the generalised hypopigmentation in him. Parents were heterozygous carriers for both the variants.

**Family 9**

A three years old male (III-3) was evaluated for heterochromia iris, telecanthus, white forelock, bilateral profound to severe hearing loss and hypopigmented patches of skin on the forehead, chest and upper limbs. He was clinically diagnosed to have Waardenburg syndrome type 1. His father (II-2) and paternal grandmother (I-2) had heterochromia iridis. His mother (II-3) was noted to have white forelock, telecanthus and hypopigmented patches on the skin. Sanger sequencing of *PAX3* (NM_181459.3) was performed first. A novel missense variant, c.166C>T p.(Arg56Cys) was identified in the proband and mother in heterozygous state, confirming the diagnosis of Waardenburg syndrome type 1 (MIM# 193500). The same variant was present in the proband’s elder brother who was clinically unaffected and thus showed non penetrance for Waardenburg syndrome. This variant was not found in his father and paternal grandmother. Due to the presence of heterochromia iridis in father and paternal grandmother, ES of the father was done. A novel single base pair deletion, c.1047delC p.(Val350PhefsTer36) in *EDNRB* (NM_001201397.1) in heterozygous state was identified, causing Waardenburg syndrome type 4A (MIM# 277580). The same variant was present in the paternal grandmother and the proband. None of them had features of Hirschsprung disease.

**Family 10**

A 17 years old male (III-2) born to non-consanguineous parents, was evaluated for telecanthus, heterochromia iridis, white forelock and bilateral hand contractures. He was clinically diagnosed to have Waardenburg syndrome type 3 (MIM# 277580). There was a family history of hearing loss in both parents, paternal uncle and paternal grandmother (II-1, II-2, II-3, I-2). There was a history of heterochromia iridis and hearing loss in paternal grandmother (I-2). Sanger sequencing of *PAX3* (NM_181459.3) detected a novel nonsense variant, c.829C>T p.(Gln277Ter) in heterozygous state, confirming the diagnosis of Waardenburg syndrome type 3 (MIM# 148820) in the proband. His mother, who had hearing loss, did not have this variant. Father’s sample was not available for testing. *GJB2* (NM_004004.5) sequencing for the mother was done, since it was the most common gene associated with hearing loss. A known nonsense variant, c.71G>A p.(Trp24Ter) causing and deafness, autosomal recessive 1A (MIM# 220290) in a homozygous state was identified in her (7). The same variant was found in a homozygous state in the proband as well.

**Family 11**

Three-years-six months-old male (IV-3), born to third degree consanguineous parents was evaluated for seizures and neuroregression. He had two elder siblings, who expired due to similar complaints. WES in the proband identified a novel homozygous missense variant, c.205C>G p. (Leu69Val) in *KCTD7* (NM_153033.5). The variant was observed in homozygous state in his similarly affected female sibling as well, thus confirming the diagnosis of epilepsy, progressive myoclonic 3, with or without intracellular inclusions (MIM# 611726). Parents were heterozygous carriers for this variant. A known *de novo* missense variant, c.726C>A, p.Ser242Arg in heterozygous in *MEFV* (NM_000243.3) was identified confirming a second diagnosis of autosomal dominant neutrophilic dermatosis, acute febrile (MIM# 608068) (8). The family was lost to follow up. Hence the clinical significance of the variant in *MEFV* could not be ascertained.

**Family 12**

A 17-months-old female (IV-1), first born to third-degree consanguineous parents was evaluated for global developmental delay and seizures. She was born at full term following an uneventful pregnancy with a birth weight of 2.2 kg (-2 SD). She attained head control by the age of thirteen-months, social smile by twelve-months and babbling by eight months of age. She had the first episode of unprovoked seizure at 2 months of age and second episode of seizure at 8 months of age preceded by a febrile illness. On examination, her height was 80 cm (normal), and her head circumference was 41.5 cm (-5.4SD). She had microcephaly, small and bulbous nose, full cheeks, thick vermillion, roving eye movements, opisthotonic posturing and spasticity of all four limbs. Electroencephalography at 9 months of age showed a mild degree of generalized non-specific dysfunction of electrical activity. MRI brain at 9 months was suggestive of delayed myelination, cystic changes in the fronto-temporal white matter and moderate cerebral parenchymal volume reduction with consequent thinning of corpus callosum. Singleton WES identified a known missense variant, c.205C>T p.(Arg69Trp) in exon 2 of *RNASEH2C* (NM_032193.3) in homozygous state and novel missense variants, c.[2086G>C];[3695C>A] p.[(Gly696Arg)];[(Pro1232Gln)] in exon 17 and 29 respectively in *VARS1* (NM_006295.3) in compound heterozygous state (9-11). These findings suggested dual diagnosis of Aicardi-Goutières syndrome 3 (MIM# 610329) and neurodevelopmental disorder with microcephaly, seizures, and cortical atrophy (MIM# 617802) in the proband. Segregation analysis revealed that her parents were heterozygous carriers for both these variants.

**Family 13**

One year nine months old female, second born to a non-consanguineously married couple was evaluated in view of global developmental delay, seizures, sensorineural hearing loss, microphthalmia, hypopigmented patches on forehead, abdomen and hyperpigmented patch on right leg. She was born at term via lower segment caesarean section with a birth weight of 3.5 kg (1.6 SD). She cried immediately at birth, did not open eyes till one month of age. Partial neck holding is achieved as of now (currently, one year nine months old) and she cannot sit independently with no other milestones achieved. Electroencephalogram at one year nine months of age was suggestive of electroclinical seizures of right temporal onset consistent with right focal status epilepticus with encephalopathy and she is currently on medication for the same. Magnetic resonance imaging of brain showed developmental venous malformation. Audiometry testing at the time of birth was suggestive of sensorineural hearing loss. Singleton WES was performed for her. A known *de novo* stopgain variant, c.538C>T, p.Arg180Ter in exon 3 of *MECP2* (NM_001110792.2) was identified in her(12). On copy number variant (CNV) analysis from WES data, we identified a 470 kb deletion involving the complete *KIT* gene which is known to cause piebaldism (MIM# 172800)*.* To validate this deletion and mark the exact breakpoints, we performed a chromosomal microarray (750k array, Affymetrix) for her and it revealed a 24.19 Mb heterozygous deletion at 4q12 region with the involvement of at least 22 genes including *KIT, SGCB, CHIC2, GSX2, PDGFRA, KDR, SRD5A3, TMEM165, CEP135, SPINK2, REST, IGFBP7, TECRL, GNRHR, UGT2B17, MUC7, AMTN, AMBN, ENAM, SLC4A4, ADAMTS3, ALB, AFP.* Thus, she is likely to be affected with Rett syndrome (MIM# 312750) and microdeletion at 4q12 region involving *KIT* associated with piebaldism.

**Family 14**

A 11-months-old male (III-3), first born to non-consanguineous parents, presented with a complaint of delayed development since 5 months of age and episodes of blank stare since 4 months of age. He was born preterm at 8 months of pregnancy with a birth weight of 2.6kg (normal). He cried immediately after birth. There was a history of jerky movements at birth, episodes of upward vacant stare and weak cry. He attained palmar grasp at 7 months of age and object transfer at 8 months of age. On examination at 10 months, he had not attained neck holding, was unable to recognize mother, social smile was present and there was no stranger anxiety. His height was 72cm (normal) and head circumference was 41cm (-6 SD). He had hypotonia, metopic prominence, orofacial and limbs dyskinetic movements, hyperactivity, microcephaly, and was unable to maintain eye contact. He did not have muscle hypertrophy. His reflexes were elicitable. Partial neck holding was attained at 11 months of age. Hearing, ophthalmological and EEG results were normal. Karyotyping performed at 6 months revealed a normal male karyotype (46, XY). MRI brain performed at 6 months of age showed mild cerebral atrophy, prominent lateral ventricles, thinning of corpus callosum and mild delay in myelination. CPK level at one year was 63U/L (20-200U/L). Trio WES performed for the family revealed the presence of a novel *de novo* missense variant c.316T>G p.(Cys106Gly) in exon 2 of *KCNQ2* (NM_172107.4) causing autosomal dominant developmental and epileptic encephalopathy 7 (MIM# 613720). Chromosomal microarray, performed earlier as a first-tier testing at an external laboratory, revealed the presence of a hemizygous pathogenic 290kb deletion at cytoband Xp21.1 spanning *DMD* gene. These findings were further confirmed using multiplex-ligation dependent amplification (MLPA) which showed hemizygous deletion of exon 44 and exon 45 in *DMD* suggesting a likely diagnosis of Becker muscular dystrophy (MIM# 300376).

**Supplementary Figure 1**


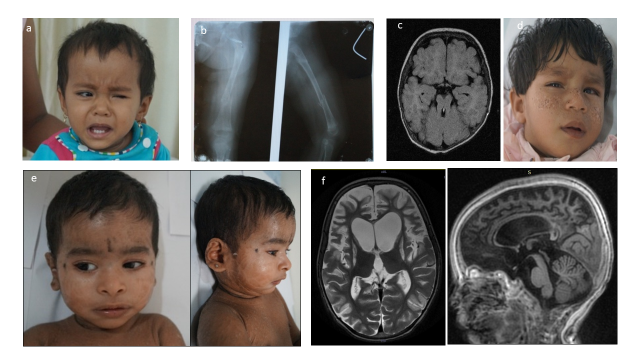


Microphthalmia (a), reduced bone density and fractures of femur in proband in family 1, molar tooth sign on magnetic resonance image of brain in III-1 in family 3 (c), photosensitive rash on face in III-2 in family 3 (d), ichthyosis in proband in family 6 (e) and MRI brain showing bilateral hyperintense lesions in both putamen with liquefaction and also in parasagittal and precentral gyri symmetrically in both frontal lobes in IV-1 in family 7

**Supplementary Figure 2: Punnett square**

1. **Two autosomal recessive disorders**


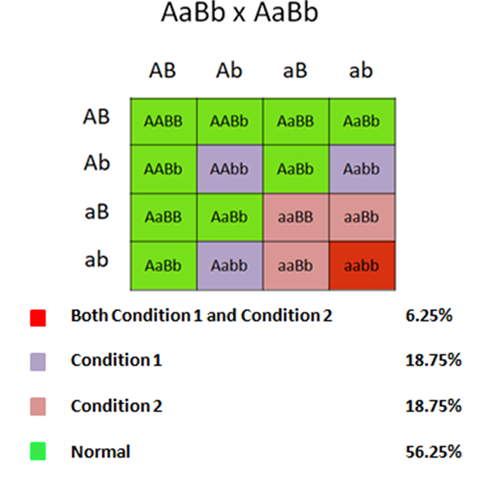


**B. Two autosomal dominant disorders**


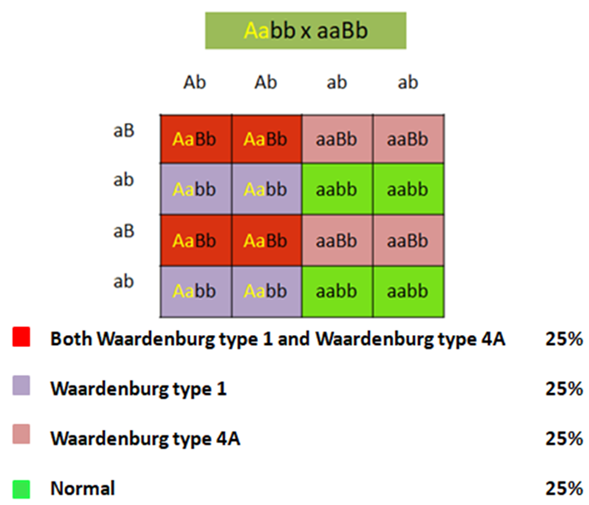


**C. Three autosomal recessive disorders**


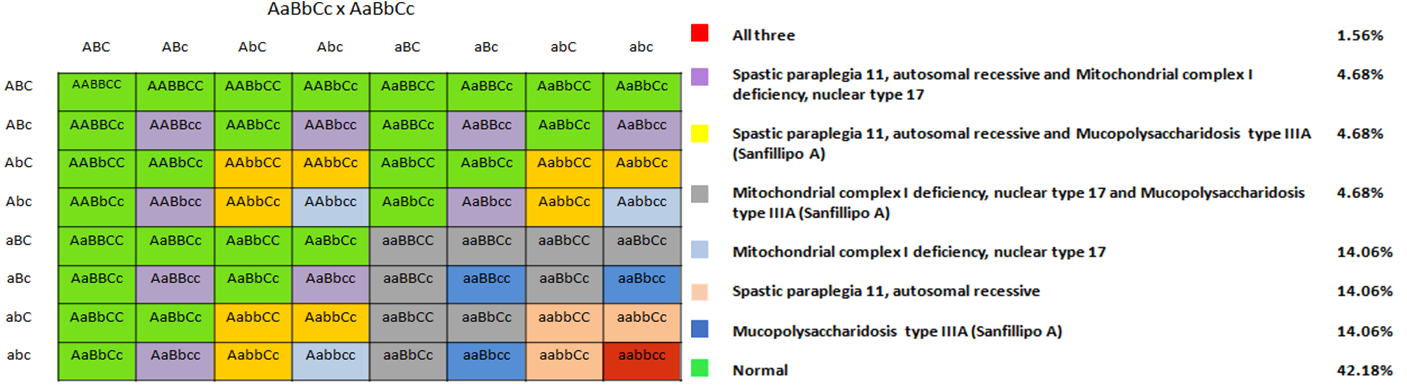


**References:**

1. Tsujino S, Servidei S, Tonin P, Shanske S, Azan G, DiMauro S. Identification of three novel mutations in non-Ashkenazi Italian patients with muscle phosphofructokinase deficiency. Am J Hum Genet. 1994;54(5):812-9.

2. Kroes HY, Monroe GR, van der Zwaag B, Duran KJ, de Kovel CG, van Roosmalen MJ, et al. Joubert syndrome: genotyping a Northern European patient cohort. Eur J Hum Genet. 2016;24(2):214-20.

3. Messaoud O, Rekaya MB, Ouragini H, Benfadhel S, Azaiez H, Kefi R, et al. Severe phenotypes in two Tunisian families with novel XPA mutations: evidence for a correlation between mutation location and disease severity. Arch Dermatol Res. 2012;304(2):171-6.

4. Eng B, Nakamura LN, O'Reilly N, Schokman N, Nowaczyk MM, Krivit W, et al. Identification of nine novel arylsulfatase a (ARSA) gene mutations in patients with metachromatic leukodystrophy (MLD). Hum Mutat. 2003;22(5):418-9.

5. Dahlqvist J, Klar J, Hausser I, Anton-Lamprecht I, Pigg MH, Gedde-Dahl T, Jr., et al. Congenital ichthyosis: mutations in ichthyin are associated with specific structural abnormalities in the granular layer of epidermis. J Med Genet. 2007;44(10):615-20.

6. Chaki M, Sengupta M, Mondal M, Bhattacharya A, Mallick S, Bhadra R, et al. Molecular and functional studies of tyrosinase variants among Indian oculocutaneous albinism type 1 patients. J Invest Dermatol. 2011;131(1):260-2.

7. Kelsell DP, Dunlop J, Stevens HP, Lench NJ, Liang JN, Parry G, et al. Connexin 26 mutations in hereditary non-syndromic sensorineural deafness. Nature. 1997;387(6628):80-3.

8. Masters SL, Lagou V, Jeru I, Baker PJ, Van Eyck L, Parry DA, et al. Familial autoinflammation with neutrophilic dermatosis reveals a regulatory mechanism of pyrin activation. Sci Transl Med. 2016;8(332):332ra45.

9. Hebbar M, Kanthi A, Shrikiran A, Patil S, Muranjan M, Francis F, et al. p.Arg69Trp in RNASEH2C is a founder variant in three Indian families with Aicardi-Goutieres syndrome. Am J Med Genet A. 2018;176(1):156-60.

10. Nishimura T, Baba M, Ogawa S, Kojima K, Takita T, Crouch RJ, et al. Characterization of six recombinant human RNase H2 bearing Aicardi-Goutieres syndrome causing mutations. J Biochem. 2019;166(6):537-45.

11. Vogt J, Agrawal S, Ibrahim Z, Southwood TR, Philip S, Macpherson L, et al. Striking intrafamilial phenotypic variability in Aicardi-Goutieres syndrome associated with the recurrent Asian founder mutation in RNASEH2C. Am J Med Genet A. 2013;161A(2):338-42.

12. Gauthier J, de Amorim G, Mnatzakanian GN, Saunders C, Vincent JB, Toupin S, Kauffman D, St-Onge J, Laurent S, Macleod PM, Minassian BA, Rouleau GA. Clinical stringency greatly improves mutation detection in Rett syndrome. Can J Neurol Sci. 2005 Aug;32(3):321-6. doi: 10.1017/s0317167100004200. PMID: 16225173.
